# Dectin-1-Mediated Production of Pro-Inflammatory Cytokines Induced by Yeast β-Glucans in Bovine Monocytes

**DOI:** 10.3389/fimmu.2021.689879

**Published:** 2021-05-28

**Authors:** Ana R. V. Pedro, Tânia Lima, Ricardo Fróis-Martins, Bárbara Leal, Isabel C. Ramos, Elisabete G. Martins, Ana R. J. Cabrita, António J. M. Fonseca, Margarida R. G. Maia, Manuel Vilanova, Alexandra Correia

**Affiliations:** ^1^Immunobiology Group, i3S – Instituto de Investigação e Inovação em Saúde, Universidade do Porto, Porto, Portugal; ^2^Laboratório de Imunologia, DIMFF, ICBAS – Instituto de Ciências Biomédicas Abel Salazar, Universidade do Porto, Porto, Portugal; ^3^LAQV, REQUIMTE, ICBAS – Instituto de Ciências Biomédicas Abel Salazar, Universidade do Porto, Porto, Portugal; ^4^Laboratório de Imunogenética, DPIM, ICBAS – Instituto de Ciências Biomédicas Abel Salazar, Universidade do Porto, Porto, Portugal; ^5^UMIB, Instituto de Ciências Biomédicas Abel Salazar, Universidade do Porto, Porto, Portugal; ^6^Animal Nutrition Division, Cooperativa Agrícola de Vila do Conde, Vila do Conde, Portugal; ^7^ADM Portugal, SA, Murtede, Portugal; ^8^EPIUnit, Instituto de Saúde Pública, Universidade do Porto, Porto, Portugal; ^9^Department of Veterinary Medicine, Escola Universitária Vasco da Gama, Coimbra, Portugal

**Keywords:** dectin-1, β-glucans, bovine, monocytes, cytokines, siRNA, CLEC7A

## Abstract

Yeast-derived products containing β-glucans have long been used as feed supplements in domesticated animals in an attempt to increase immunity. β-glucans are mainly recognized by the cell surface receptor CLEC7A, also designated Dectin-1. Although the immune mechanisms elicited through Dectin-1 activation have been studied in detail in mice and humans, they are poorly understood in other species. Here, we evaluated the response of bovine monocytes to soluble and particulate purified β-glucans, and also to Zymosan. Our results show that particulate, but not soluble β-glucans, can upregulate the surface expression of costimulatory molecules CD80 and CD86 on bovine monocytes. In addition, stimulated cells increased production of IL-8 and of *TNF, IL1B*, and *IL6* mRNA expression, in a dose-dependent manner, which correlated positively with *CLEC7A* gene expression. Production of IL-8 and *TNF* expression decreased significantly after *CLEC7A* knockdown using two different pairs of siRNAs. Overall, we demonstrated here that bovine monocytes respond to particulate β-glucans, through Dectin-1, by increasing the expression of pro-inflammatory cytokines. Our data support further studies in cattle on the induction of trained immunity using dietary β-glucans.

## Introduction

Immune modulation by natural compounds has long been studied in domesticated animals such as poultry, fish, and livestock, to enhance immunity and improve animal welfare and wellbeing, ultimately reducing the incidence of disease and the overuse of pharmaceutical compounds, such as antibiotics. Dietary supplementation with yeasts (1–6) and yeast-derived compounds, such as mannan-oligosaccharides (MOS) and β-glucans (1, 4–7), is one of the most used strategies to enhance immunity in domesticated animals. β-glucans are naturally occurring polymers present in the cell wall of fungi, bacteria, algae, and plants. Yeast β-glucans are usually composed of linear molecules of D-glucose units linked by β-1,3 glycosidic bonds with β-1,6 branching (8, 9). β-glucans are recognized by immune cell surface pattern recognition receptors (PRR) such as C-type lectin domain family 7 member A (CLEC7A), also designated as Dectin-1, complement receptor-3 (CR3), scavenger receptors, and lactosylceramide (10). In addition, Toll-like receptor (TLR)-2 and TLR-6 can synergistically contribute to the recognition and elicited biological effects of particulate β-glucans, such as Zymosan (11–14). Biological activities of β-glucans depend on their recognition and downstream cell signalling, which in turn depend largely on the structure, conformation, and physical properties of the different β-glucans (9, 15). Although both particulate and soluble β-glucans bind Dectin-1, only the particulate form can induce Dectin-1 signalling and generate a “phagocytic synapse” (16). Activation of Dectin-1 triggers an intracellular signalling cascade eliciting phagocytosis, production of cytokines, and reactive-oxygen species (ROS) (14–19).

The immune recognition of β-glucans and elicited response has been extensively studied in mice and humans at mechanistic level (15, 20, 21). However, in other species, including cattle, the effects of β-glucans on the immune system are mainly supported by observational reports and *in vivo* studies (22–25). A homologous transcript for human Dectin-1 has been described in bovines (boDectin-1) (26). However, β-glucan recognition and its effects on bovine leukocytes were not fully elucidated.

Bovine monocytes express *CLEC7A* (26) and this cell type is the most used in innate immune memory studies in other species (27, 28). Here, the response of bovine monocytes to soluble and particulate β-glucans, and to β-glucan-containing particles (Zymosan) was assessed. Our results show that only the particulate β-glucan forms trigger the production of pro-inflammatory cytokines, and implicate boDectin-1 in this effect.

## Materials and Methods

### Isolation of Bovine Peripheral Blood Monocytes

Bovine blood from Holstein-Friesian cattle was obtained at a local commercial slaughterhouse (PEC Nordeste – Indústria de Produtos Pecuários do Norte, Penafiel, Portugal) and licensed by National competent authority, Direção Geral de Alimentação e Veterinária, under a by-product handling authorization (N.12.006.UDER). There was no intervention on the animals for research purposes, since blood was collected during bleeding/slaughter of animals for human consumption. Blood was collected from jugular and carotid veins to BD Vacutainer^®^ lithium heparin tubes (BD, Franklin Lakes, NJ, USA) and peripheral blood CD14^+^ monocytes were obtained as previously described, with minor modifications (29). Briefly, whole blood was diluted 1:2 with Dulbecco’s phosphate-buffered saline (DPBS) and density gradient centrifuged on Histopaque^®^-1077 (both from Sigma-Aldrich) at 1200 × g for 15 min in SepMate™ PBMC isolation tubes (Stemcell™ Technologies, Vancouver, BC, Canada). Peripheral blood mononuclear cells (PBMC) were then washed with DPBS by centrifugation at 400 × g for 10 min and CD14^+^ cells were isolated with anti-human CD14 MicroBeads, according to manufacturer’s instructions (Miltenyi Biotec, Bergisch Gladbach, Germany). Peripheral blood CD14^+^ monocytes were washed with DPBS by centrifugation at 300 × g for 10 min and resuspended at 2 × 10^6^ cells/mL in complete RPMI medium - RPMI-1640 Medium (Sigma-Aldrich) supplemented with 10% FBS (Biowest, Nuaillé, France), 50 µM β-mercaptoethanol (Merck, Darmstadt, Germany), 100 U/mL penicillin, 100 µg/mL streptomycin, 4 mM L-glutamine and 10 mM HEPES (all from Sigma-Aldrich). Purity of CD14^+^ cells exceeded 90%, as evaluated by flow cytometry using an anti-sheep CD14 mAb (clone VPM65, Bio-Rad, Hercules, CA, USA) that cross reacts with bovine, conjugated with DyLight^®^ 405 Conjugation Kit (Abcam, Cambridge, UK).

### Cell Culture and Stimulation With β-Glucans

Bovine CD14^+^ monocytes were plated at 2 × 10^5^ cells/well in flat-bottom 96-well culture plates. Stimulation was done with a pure soluble β-glucan preparation (WGP^®^-Soluble), a purified insoluble preparation of *Saccharomyces cerevisiae* lacking TLR activity, composed mainly of β-1,3-glucans (WGP^®^-Dispersible), and an insoluble preparation of *S. cerevisiae* cell wall (Zymosan), described to have Dectin-1- and TLR2/6-stimulatory activity (all from InvivoGen, San Diego, CA, USA). β-glucans, labelled endotoxin-free (endotoxin level below 0.001 EU/μg), were prepared according to manufacturer’s instructions. All assays were performed using sterile, pyrogen-free material. Cells were cultured with 10, 50 and 100 µg/mL of WGP-Soluble, WGP-Dispersible or Zymosan. Cells cultured with 1 µg/mL of *Escherichia coli* lipopolysaccharide (LPS, strain O111:B4; Sigma-Aldrich) or Pam3CSK4 (P3C; InvivoGen) were used as positive controls. A kinetic cytokine mRNA expression analysis was performed in cells cultured for 8, 16, and 24 h at 37°C and 5% CO_2_ to define the time point for sequent analyses. Cytokine production, mRNA expression, and lactate dehydrogenase (LDH) release, were assessed in cells cultured for 24 h at 37°C and 5% CO_2_. Cell surface activation markers were assessed by flow cytometry in 8 and 16 h bovine monocyte cultures. Non-stimulated cells were always used as negative controls.

### HEK-Blue™ hDectin-1b Reporter Cell Line Culture and Stimulation with β-Glucans

Hek-Blue hDectin-1b cells (InvivoGen) were grown in DMEM medium with 4.5 g/L glucose (Sigma-Aldrich), 10% heat inactivated Fetal Bovine Serum Premium (FBS) (Biowest), 2 mM L-glutamine, 100 U/mL penicillin, 100 μg/mL streptomycin (all from Sigma-Aldrich), 100 μg/mL Normocin™ and 1 µg/mL puromycin (both from InvivoGen) in vented T75 flasks. When cells reached 80% confluency, they were re-seeded at 5 × 10^4^ cells/well in flat-bottom 96 well-culture plates and stimulated with 10, 50 and 100 µg/mL of WGP^®^-Soluble, WGP^®^-Dispersible, or Zymosan in HEK-Blue™ Detection medium for 16 h. Substrate hydrolysis by secreted alkaline phosphatase (SEAP), upon activation of the receptor, was assessed at 620-655 nm according to manufacturer’s instructions in a BioteK™ µQuant Microplate Reader using Biotek™ Gen5™ Data Collection and Analysis Software (Thermo Fisher Scientific, Waltham, MA, USA).

### Cell Viability Assays

LDH release was quantified in cell culture supernatants using CyQUANT™ LDH Cytotoxicity Assay kit, according to manufacturer’s instructions (Invitrogen, Waltham, MA, USA).

### Cytokine Production

Cytokine levels were assessed in cell culture supernatants by sandwich ELISA. Assessment of bovine Tumor Necrosis Factor Alpha (TNF-α) and bovine Interleukin (IL)-6 was done using Bovine TNF-alpha and Bovine IL-6 DuoSet ELISA kits (R&D Systems, Minneapolis, MN, USA) according to the manufacturer’s protocol with a minor modification: 1% molecular grade bovine serum albumin (BSA, Albumine Bovine Fraction V, NZYTech, Lisbon, Portugal) in DPBS was used as reagent diluent, instead of 5% Tween 20 in DPBS. Bovine IL-8 (Bovine IL-8 [CXCL8] ELISA development kit, Mabtech AB, Nacka Strand, Sweden) was quantified according to manufacturer’s instructions. Detection limits: 8 pg/mL for IL-8 and 125 pg/mL for TNF-α and IL-6. Bovine IL-1β was quantified using the IL-1 beta Bovine Uncoated ELISA Kit (Invitrogen), according to manufacturer’s instructions. Detection limit <31.3 pg/mL. Bovine IL-10 was assessed in cell culture supernatants using an in-house ELISA kit, following the standard procedure of Mabtech ELISA Bovine IL-8 kit. Briefly, Nunc Maxisorp™ plates were coated with 1 µg/mL anti-bovine IL-10 mAb (clone CC318; Bio-Rad) in PBS and incubated overnight at 4°C. A nine-point standard curve from 2000 to 8 pg/mL was done using Recombinant Bovine Interleukin-10 (Bio-Rad). Cell supernatants and standards were incubated at room temperature for 2 h, followed by incubation for 1 h with the detection antibody at 0.5 µg/mL (mouse anti-Bovine Interleukin-10:Biotin; clone CC320; Bio-Rad) and 1 h with Mabtech’s streptavidin-HRP, according to manufacturer’s instructions. Detection limit: 8 pg/mL. Only samples above detection limits were used for comparison.

### Bovine Dectin-1 Knockdown Assays (Small Interference RNA)

The following small interfering RNAs (siRNA) were designed by Custom siRNA Design Service (Merck) to target both isoforms of bovine Dectin-1 and achieve the knockdown of this receptor: siRNA #1 sense AUG AAG AUG GAU AUA CUC A dTdT, antisense UGA GUA UAU CCA UCU UCA U dTdT; siRNA #2 sense UGA GGA UAG CUG UUA UCU A dTdT, antisense UAG AUA ACA GCU AUC CUC A dTdT; siRNA #3 sense GAG GAU AGC UGU UAU CUA U dTdT, antisense AUA GAU AAC AGC UAU CCU C dTdT (all from Sigma-Aldrich). Transfection procedure was performed for 4 h in serum-free X-VIVO™ 15 haematopoietic medium (Lonza, Basel, Switzerland) with ScreenFect^®^siRNA transfection reagent, according to manufacturer’s instructions (ScreenFect GmbH, Eggenstein-Leopoldshafen, Germany), and 300 nM of siRNA duplexes or siRNA negative control (MISSION^®^ siRNA Universal Negative Control #1, Sigma-Aldrich). After transfection, cells were washed with non-supplemented RPMI-1640 medium and incubated for 24 h at 37°C and 5% CO2 with WGP^®^-Soluble, WGP^®^-Dispersible and Zymosan^®^ at 50 µg/mL in RPMI medium or with medium alone. Supernatants were collected to assess IL-8 production and cells were preserved in NZYol reagent (NZYTech, Lisboa, Portugal) to assess *CLEC7A, TNF, IL1B, IL6*, and *IL10* mRNA expression.

### RNA Extraction and cDNA Synthesis

Total RNA was obtained using NZYol according to manufacturer’s protocol, with minor modifications. Bovine monocytes were lysed with 200 µL NZYol and incubated with 0.1 µg/mL RNA-grade Glycogen (Thermo Fisher Scientific). Each sample was incubated with 80 µL chloroform for phase separation and 200 µL isopropanol was added for RNA precipitation. The precipitated RNA was washed with 70% ethanol and resuspended in 5 µL RNase-free water. Synthesis of first-strand cDNA was done in an Applied Biosystems^®^ 2720 Thermal Cycler (Thermo Fisher Scientific) at 25°C for 10 min, 50°C for 30 min, and 85°C for 5 min using NZY First-Strand cDNA Synthesis Kit, according to manufacturer’s instructions (NZYtech). Samples were kept at -20°C. Negative controls using RNA samples for cDNA synthesis without reverse transcriptase (no RT control), and with no added template (no template control) were also included for all primer pairs.

### Real-Time qPCR

Primers for β2 microglobulin (*B2M*), *CLEC7A*, *IL1B*, and *IL6* were designed using Primer-BLAST web tool developed by NCBI (30). Primers for *TNF* and *IL10* were previously designed (31, 32). Sequences of each primer and expected amplicon sizes are detailed in **Table 1**. Primers targeting *CLEC7A* were designed to both short and long isoforms. Determination of *TNF*, *IL1B, IL6*, *IL10* and *CLEC7A* mRNA levels was performed in a CFX96™ Real-Time PCR Detection System (Bio-Rad), using NZYSpeedy qPCR Green Master Mix (2×) ROX plus (NZYTech). *B2M* and MARVEL domain containing 1 (*MARVELD1*), already used as reference genes in bovine gene expression studies, were used for mRNA normalization (33–35). Reaction was performed in low profile, non-skirted, 96-well PCR plates (Thermo Fisher Scientific) containing 5 µL Master Mix, 1 µL cDNA, 3.6 µL H2O and 0.2 mM of specific forward and reverse primers (all from Sigma-Aldrich). PCR program was as follows: denaturation for 5 min at 95°C followed by 40 cycles at 95°C for 5 s and 62°C for 20 s for amplification. Gene expression values were analyzed by the comparative threshold cycle method using the formula 2^-(CT gene of interest - CT housekeeping gene)^ (36). *CLEC7A* PCR products were run in 1.5% (w/v) Tris-acetate-EDTA (TAE) agarose gel electrophoresis to confirm amplicon size. Bands were visualized in a Syngene™ NuGenius Gel Documentation System, excised from the gel and purified using NZYGelpure columns (NZYTech) following manufacturer’s instructions. PCR products and DNA purified from excised gel bands were Sanger sequenced to confirm primer specificity.

**Table 1:** List of primers used for quantitative real-time PCR.

Gene	Primer^b^ Sequence 5’-3’	Amplicon Size (bp)	GeneBank Accession Number or Reference
*CLEC7A*^a^	F: TGCTGTGACTCTGGGCATTT	235 Long 97 Short	AY937383.1 AY937382.1
	R: CCAGTTAGGGGGACAAGAGC		
*TNF*	F: CCAGAGGGAAGAGCAGTCCC	114	(33)
	R: TCGGCTACAACGTGGGCTAC		
*IL10*	F: AGAACCACGGGCCTGACAT	151	(34)
	R: AGCTCACTGAAGACTCTCTTCACCTT		
*IL6*	F: CCTGAAGCAAAAGATCGCAGA	204	NM_173923.2
	R: ATGCCCAGGAACTACCACAA		
*MARVELD1*	F: GGCCAGCTGTAAGATCATCACA	100	(36)
	R: TCTGATCACAGACAGAGCACCAT		
*B2M*	F: AAGTGGGATCGAGACCTGTAA	191	NM_173893.3
	R: GGACATGTAGCACCCAAGGTAA		
*IL1B*	F: AAACTCCAGGACAGAGAGCAAAA	126	NM_174093.1
	R: CTCTCCTTGCACAAAGCTCATG		

a Amplicon Size (bp) of short and long isoforms.

b Primer direction: F, Forward; R, Reverse.

### Flow Cytometry

Since the commercially existing labelled antibodies for the bovine species are available in limited fluorophore diversity, we conjugated mouse monoclonal anti-bovine MHC class II DR (clone CC108, Bio-Rad) antibody with peridinin-chlorophyll protein-cychrome 5.5 (PerCP-Cy5.5) with LYNX Rapid PerCP-Cy5.5 Antibody Conjugation Kit (Bio-Rad), according to manufacturers’ instructions, and used it at 1:200 to allow multiparametric simultaneous analysis with the monoclonal antibodies mouse anti-bovine CD80 conjugated with R-Phycoerythrin (RPE) (clone IL-A159, Bio-Rad) and mouse anti-bovine CD86 conjugated with Fluorescein isothiocyanate (FITC) (clone IL-A190, Bio-Rad), both used at 1:50. All the antibodies were previously titrated to determine the optimal concentration for bovine monocyte staining. A fixable viability dye (FVD) was included before surface antibody staining to exclude dead cells from the analysis. For that, cells were incubated with eFluor^®^ 506 Fixable Viability Dye (eBioscience, San Diego, CA, USA) diluted at 1:1000 in DPBS for 15 min at 4°C. After washing cells with DPBS, cells were incubated in 2% mouse serum in FACS Buffer (1% BSA in DPBS) for 15 min at 4°C in the dark before antibody staining to minimize nonspecific binding. A mix containing all antibodies was added to samples that were incubated for 25 min at 4°C in the dark. Cells were washed with FACS Buffer and analyzed by flow cytometry. Single stainings using UltraComp beads (eBioscience) or/and cells were used for compensation. Fluorescence minus one controls (FMO) of each antibody and FVD were used for gating purposes. Data were acquired in a BD CantoII™ equipment (BD Biosciences) and analyzed with FlowJo version 10.6.2. (FlowJo LLC, Ashland, OR, USA).

### Bovine Dectin-1 Staining

Bovine monocytes and Hek-Blue hDectin-1b cells were incubated with monoclonal mouse anti-Human Dectin-1/CLEC7A antibody (Clone 259931, R&D Systems) at 10 µg/mL for 1 h at 4°C followed by incubation with the anti-mouse IgG (H+L) F(ab’)2 Fragment conjugated with Alexa Fluor^®^ 488 (Cell Signaling Technology, Danvers, MA, USA) at 1:200 for 30 min at 4°C in the dark. Cell staining was evaluated by flow cytometry in a BD CantoII™ cytometer (BD Biosciences) and analyzed with FlowJo version 10.6.2.

Imaging of Dectin-1 on the surface of bovine monocytes and HEK-Blue™ hDectin-1b cells was done in cells stained as before. Nuclei were stained with DAPI and samples were plated in 8 well microscopy chamber plates (Ibidi, Gräfelfing, Germany) and observed in a laser scanning confocal microscope Leica TCS SP5 II system (Leica DMI6000-CS microscope with LAS AF Software, Leica Microsystems, Wetzlar, Germany). Images were obtained with a HC PL APO CS 40x/1.10 CORR Water objective. Cells were observed using 405 nm and 488 nm lasers, in the xy plane.

### Statistical Analysis

Log transformations were applied to cytotoxicity, cytokine production, mRNA expression, siRNA assays, and flow cytometry data following a lognormal distribution. All data were analyzed using the MIXED Procedure of the SAS software (Version 9.1, SAS Institute Inc., Carry, NC, USA). The model included the fixed effect of treatment (Medium, WGP-Soluble, WGP-Dispersible, and Zymosan), the random effect of animal blood donor and the random residual error. The Tukey-Kramer’s post-hoc test was used to compare means of cytotoxicity, cytokine production, cytokine mRNA expression, siRNA assays, and flow cytometry data, whereas for hDectin-1b activity in HEK-Blue™ hDectin-1b cells data were used the Dunnett’s multiple comparisons test (SAS software). Cytokine levels or mRNA expression were expressed as Log fold changes to the respective values of control (Medium) samples. The Pearson correlations between bovine Log *CLEC7A* mRNA expression of non-stimulated samples and Log Fold change cytokine production or cytokine mRNA expression, for each stimulus, were estimated using the CORR procedure of the SAS software. Results were considered statistically significant if *P*<0.05 and a tendency if 0.05≥*P*<0.1. Graphs were constructed with the GraphPad software (Version 9.0.2, San Diego, CA, USA).

## Results

### HEK-Blue™ Cell Stimulation

The stimulatory effect of different commercial β-glucans or β-glucan-containing particles were tested in the HEK-Blue™ hDectin-1b reporter assay as a control prior to stimulation of bovine monocytes (**Supplementary Figure 1**). Soluble β-glucans did not activate hDectin-1b at any of the concentrations used, while dispersible β-glucans activated hDectin-1b at 50 and 100 µg/mL (*P*<0.01) comparatively to unstimulated cells. Zymosan significantly stimulated HEK-Blue™ hDectin-1b cells at all concentrations tested, 10 µg/mL (*P*<0.001), 50 µg/mL (*P*<0.0001) and 100 µg/mL (*P*<0.01).

### Bovine Monocyte Stimulation

**Figure 1:**
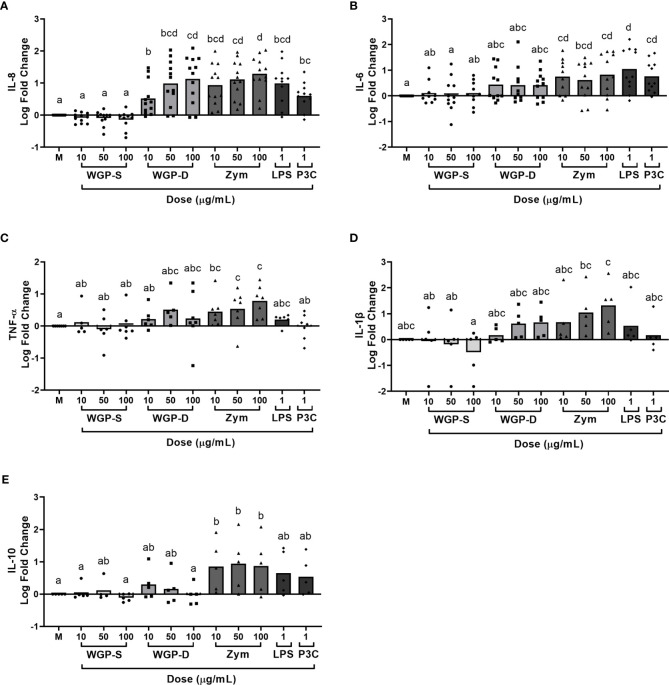
Cytokine production evaluated by ELISA in the supernatants of bovine monocytes cultured for 24 h with WGP Soluble (WGP-S), WGP Dispersible (WGP-D), Zymosan (Zym), LPS, and Pam3csk4 (P3C). Data are presented as Log fold change relative to medium (M) and represent means of 12 animals for IL-8 **(A)**, 11 animals for IL-6 **(B)**, 7 animals for TNF-α **(C)**, and 5 animals for IL-1β **(D)** and IL-10 **(E)**. Each symbol corresponds to a different animal. ^a,b,c,d^ Means with different superscript letters are significantly different (*P*<0.05).

**Figure 2:**
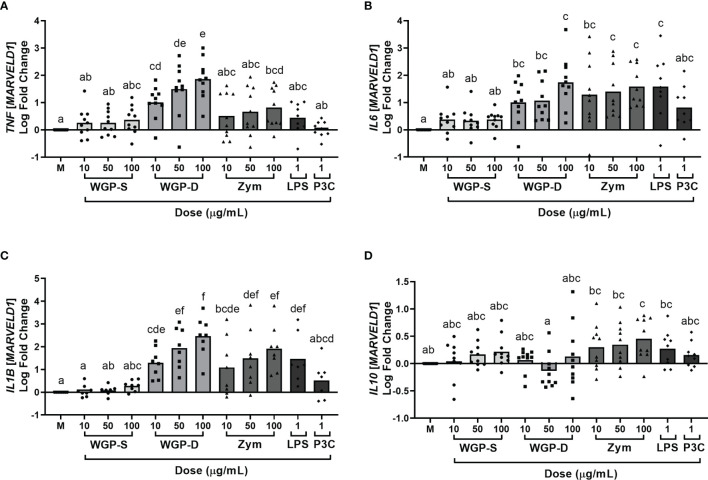
Cytokine relative mRNA expression, evaluated by RT-PCR and normalized to the mRNA expression of the reference gene *MARVELD1*, in bovine monocytes cultured for 24 h with WGP Soluble (WGP-S), WGP Dispersible (WGP-D), Zymosan (Zym), LPS, and Pam3csk4 (P3C). Data are presented as Log fold change relative to medium (M) and represent means of ten animals for *TNF* **(A)**, *IL6* **(B)**, and *IL10* **(D)**, and eight animals for *IL1B* **(C)**. Each symbol corresponds to a different animal. ^a,b,c,d,e,f^ Means with different superscript letters are significantly different (*P*<0.05).

Viability assays were performed to assure none of the stimuli were cytotoxic to bovine monocytes at the working concentrations. None of the β-glucan sources, LPS, or P3C induced statistically significant cell death when compared to unstimulated cells (**Supplementary Figure 2**). The production/expression of the pro-inflammatory cytokines IL-1β, IL-6, IL-8, and TNF-α, and of the anti-inflammatory cytokine IL-10 by bovine monocytes was evaluated after stimulation with different β-glucans, LPS or P3C. These cytokines are the most commonly assessed in β-glucan-stimulated cell studies (16, 28, 37, 38). Cells were stimulated for 8, 16, and 24 h to evaluate the kinetics of cytokine expression (**Supplementary Figure 3**). The 24 h time point was selected for further studies since it allows simultaneous cytokine protein and mRNA analysis. Cytokine production and mRNA expression were affected by treatment (*P*<0.0001 for IL-8, IL-6, TNF-α levels, and *IL1B*, *IL6* and *TNF* mRNA expression; *P*=0.0013 for IL-1β and *P*=0.0002 for IL-10 levels; *P*=0.0005 for *IL10* mRNA expression) and a dose-response effect was observed for IL-8 levels (**Figure 1**), and *IL1B*, *IL6* and *TNF* expression (**Figure 2**). WGP-Soluble treatment did not significantly affect the production or gene expression of any cytokine compared with unstimulated cells (*P*>0.05). WGP-Dispersible, Zymosan, LPS and P3C induced the production of IL-8 (**Figure 1A**). IL-6 levels (**Figure 1B**) were increased in bovine monocyte cell cultures stimulated with Zymosan, LPS and P3C. TNF-α (**Figure 1C**) and IL-10 (**Figure 1E**) levels were only increased in bovine monocytes cultured with Zymosan. A tendency for increased IL-1β production was observed in cells stimulated with Zymosan (**Figure 1D**). Expression of *TNF* (**Figure 2A**) was significantly increased in cells stimulated with WGP-Dispersible, while *IL6* (**Figure 2B**) and *IL1B* (**Figure 2C**) were overexpressed in cells stimulated with WGP-Dispersible, Zymosan, and LPS. Although there was an effect of treatment on *IL10* mRNA transcript levels (**Figure 2D**), the expression of this cytokine gene was only upregulated in cells stimulated with Zymosan at 100 µg/mL, when compared to unstimulated cells. The cytokine mRNA expression results were similar when normalization was done to *B2M* mRNA expression (**Supplementary Figure 4**).

**Figure 3:**
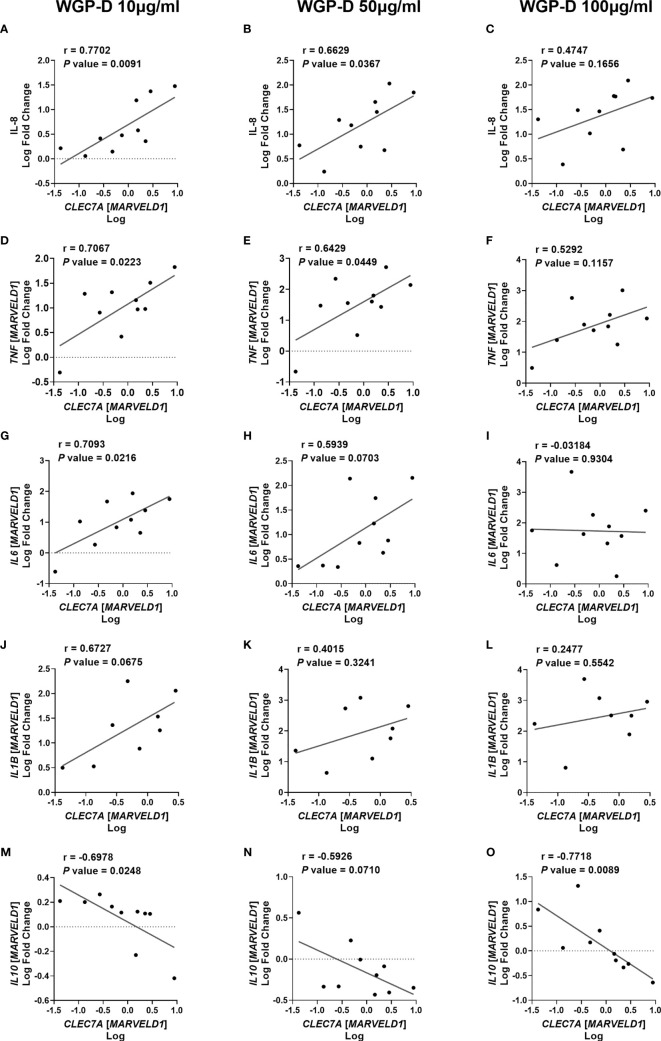
Correlations between *CLEC7A* mRNA expression and **(A–C)** IL-8 cytokine production, **(D–F)** *TNF*, **(G–I)** *IL6*, **(J–L)** *IL1B*, and **(M–O)** *IL10* mRNA expression upon stimulation with 10, 50 and 100 µg/mL of WGP Dispersible, as indicated. Results are presented as Log fold changes of each cytokine relative to medium *vs* Log *CLEC7A* mRNA. Data represent simple linear regressions, with Pearson correlation coefficients (r) and *P* values.

Since WGP-Dispersible and Zymosan stimulated cytokine production by bovine monocytes, we next examined the correlation between bovine *CLEC7A* expression of non-stimulated cells and cytokine levels or mRNA expression in response to stimulation with 10, 50 and 100 µg/mL of WGP-Dispersible and Zymosan. A positive correlation was found between *CLEC7A* mRNA expression and IL-8 concentration in the supernatants of cells stimulated with 10 µg/mL (r = 0.7702) and 50 µg/mL (r = 0.6629) (**Figures 3A, B**, respectively), but not with 100 µg/mL (**Figure 3C**) of WGP-Dispersible. *TNF* mRNA expression was positively correlated with *CLEC7A* mRNA expression at 10 µg/mL (r = 0.7067) and 50 µg/mL (r = 0.6429) (**Figures 3D, E**), but not at 100 µg/mL (**Figure 3F**). *IL6* mRNA expression was also correlated with *CLEC7A* expression in cells stimulated with WGP-Dispersible at 10 µg/mL (r = 0.7093, **Figure 3G**) and a tendency was observed in cells stimulated with 50 µg/mL (r = 0.5939, **Figure 3H**). No such correlation was observed when cells were stimulated with 100 µg/mL WGP-Dispersible (**Figure 3I**). A tendency was observed between *CLEC7A* mRNA expression and *IL1B* mRNA expression in cells stimulated with 10 µg/mL (r = 0.6672, **Figure 3J**), but not when cells were stimulated with 50 µg/mL or 100 µg/mL WGP-Dispersible (**Figures 3K, L**, respectively). Interestingly, a negative correlation was observed between *CLEC7A* and *IL10* mRNA expression in cells stimulated with 10 µg/mL (r = -0.6978, **Figure 3M**) and 100 µg/mL (r = -0.7718, **Figure 3O**) of WGP-Dispersible, and a tendency to a negative correlation in cells stimulated with 50 µg/mL (r = -0.5926, **Figure 3N**) of this β-glucan form. No correlation was found between *CLEC7A* mRNA expression and cytokine mRNA expression or production in cells stimulated with Zymosan (**Supplementary Figure 5**).

Sequencing of PCR products was done to confirm primers’ specificity (**Supplementary Figure 6**). Since more than one *CLEC7A* amplicon was amplified in each sample, PCR products were also visualized in agarose gels to confirm the molecular size of the amplicons (**Supplementary Figure 6A**). According to our data, different *CLEC7A* isoforms were expressed simultaneously in monocyte samples, since two different bands, matching the expected molecular size distribution for the two different isoform amplicons (97 bp for the short or 235 bp for the long) appeared on the electrophoresis gel. This is in line with what was previously observed and described by Willcocks et al. (26) for short and long isoforms of boDectin-1. The expression of *CLEC7A* was decreased upon treatment with WGP-Dispersible and Zymosan, but not with WGP-Soluble (**Supplementary Figure 7**). Previous reports have shown a downregulation of Dectin-1 expression on the surface of human monocyte- and mouse bone marrow-derived dendritic cells and mouse bone marrow-derived macrophages in response to particulate β-glucan stimulation (39–41). However, no mention was made therein whether *CLEC7A* gene expression was also reduced.

### Expression of MHC Class II and Costimulatory Molecules

**Figure 4:**
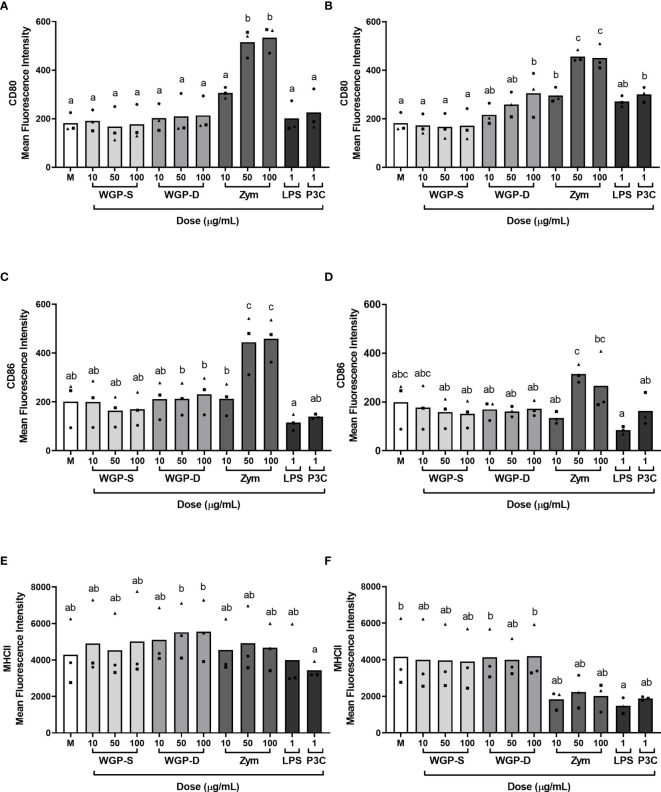
Expression of **(A, B)** CD80, **(C, D)** CD86, and **(E, F)** MHC class II molecule expression on the cell surface of bovine monocytes stimulated with WGP Soluble (WGP-S), WGP Dispersible (WGP-D), Zymosan (Zym), LPS, and Pam3csk4 (P3C) for 8 h **(A, C, E)** or 16 h **(B, D, F)**, as evaluated by flow cytometry. Results correspond to means of the mean fluorescence intensities for each analyzed molecule of three independent biological samples (each represented by squares, triangles or circles). ^a,b,c^ Means with different superscript letters are significantly different (*P*<0.05).

The expression of costimulatory (CD80 and CD86) and MHC class II molecules on the cell surface of monocytes upon stimulation with the different β-glucans or β-glucan-containing particles was evaluated by flow cytometry, as indicated in **Supplementary Figure 8**. WGP-Soluble treatment did not alter the expression of MHC class II or costimulatory molecules at any assessed time point (**Figure 4**). Zymosan treatment upregulated the expression of CD80 and CD86 at 8 h (**Figures 4A, C** respectively) at 50 and 100 µg/mL, but only the expression of CD80 remained upregulated at 16 h (**Figures 4B, D**). Cells stimulated for 16 h with 100 µg/mL WGP-Dispersible and P3C increased the expression of CD80 (**Figure 4B**). No differences in the expression of MHC class II were observed at any of these timepoints (**Figures 4E, F**), besides a decrease in LPS-treated-cells at 16 h post stimulation.

### Small Interference mRNA and Bovine Dectin-1 Knockdown

IL-8 levels and *TNF* and *IL6* expression were significantly increased in monocytes stimulated with either WGP-Dispersible or Zymosan, and their increase was found to be correlated with *CLEC7A* expression in WGP-Dispersible-treated cells. Therefore, we further investigated the role of boDectin-1 in IL-8 production and *TNF* and *IL6* expression by silencing the receptor using a siRNA approach. Three pairs of siRNA duplexes were designed to target and silence the two bovine Dectin-1 isoforms. Transfection did not affect cell viability (**Supplementary Figure 9**).

**Figure 5:**
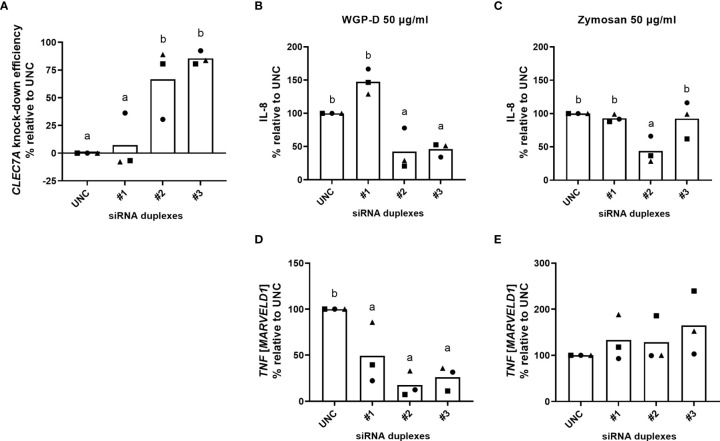
Bovine *CLEC7A* knockdown efficiency **(A)**, calculated relative to MISSION^®^ siRNA Universal Negative Control #1 (UNC) treated cells. Cells were cultured with RPMI-1640 after transfection procedure with three different siRNA duplexes (#1, #2 and #3). IL-8 production **(B, C)** and *TNF* expression **(D, E)** of cells transfected with duplexes #1, #2 and #3 and MISSION^®^ siRNA Universal Negative Control #1 (UNC) and stimulated with WGP-Dispersible **(B, D)** or Zymosan **(C, E)** at 50 µg/mL, calculated in percentual change relative to UNC transfected cells. Results correspond to means from three different animals (each one represented by squares, triangles or circles). ^a,b^ Means with different superscript letters are significantly different (*P*<0.05).

Bovine *CLEC7A* was successfully knocked down by siRNA duplex #3 (85.5%, *P*<0.01) and, to a lesser extent, by siRNA #2 (66.6%, *P*<0.05) at 300 nM (**Figure 5A**). The knockdown efficiency of this siRNA design was particularly evident in two of the samples tested (Biological samples #2 and #3, **Supplementary Table 1**). siRNA design #1 was not able to successfully knock down bovine *CLEC7A* in any of the samples tested (**Figure 5A**).

Lower IL-8 levels were found in the supernatants of cells transfected with siRNA pairs #2 and #3 upon stimulation with WGP-Dispersible at 50 µg/mL, comparatively to cells treated with medium alone (X-Vivo), ScreenFect^®^siRNA transfection reagent (Screenfect), and MISSION^®^ siRNA Universal Negative Control #1 (UNC). The percentual change of cytokine production was then calculated considering IL-8 production of Negative Control transfected cells. IL-8 production was significantly decreased (*P*<0.05) in WGP-Dispersible stimulated cells (**Figure 5B**) when transfection was performed with siRNA duplex #2 (57.6% decrease) and siRNA duplex #3 (54.1% decrease). Only siRNA duplex #2 was able to successfully reduce IL-8 production (56.4% decrease, *P*<0.05) in cells stimulated with Zymosan at 50 µg/mL (**Figure 5C**). siRNA duplex #2 led to a clear decrease in IL-8 production in two of the samples stimulated with WGP-Dispersible at 50 µg/mL (**Figure 5B**). A smaller reduction was observed in one of the samples used. The observed effect matches the low *CLEC7A* knockdown efficiency observed in this particular sample (**Figures 5A** and **Supplementary Table 1**). The expression of *TNF* in cells stimulated with WGP-Dispersible at 50 µg/mL was also significantly affected by *CLEC7A* knockdown (**Figure 5D**). No such effect was observed in Zymosan-treated cells (**Figure 5E**). Although not statistically different, the expression of *IL6* and *IL1B* was decreased upon siRNA#2 treatment in response to WGP-Dispersible (**Supplementary Figure 10**). Given the high homology of bovine and human Dectin-1, and since no bovine-specific anti-Dectin-1 antibody is available, neutralization of this receptor was attempted using an anti-human Dectin-1 mAb. The used antibody did not recognize boDectin-1 as evaluated by flow cytometry and confocal microscopy (**Supplementary Figures 11**, **12**, respectively).

## Discussion

The effects of β-glucans or β-glucan-containing products have been explored in ruminants, either *in vitro* (42–44) or *in vivo*, by oral administration (23, 24, 44–47), with the purpose of increasing immunity or response to stressors. However, the immunostimulatory effect of β-glucan-containing products, such as Zymosan, on bovine cells mostly involved the analysis of reactive oxygen and nitrogen species production by neutrophils and monocyte-derived macrophages (48–50). BoDectin-1 has been previously identified and *CLEC7A* gene expression was detected in several bovine immune cell populations, such as monocytes, monocyte-derived dendritic cells, CD4^+^ T cells, CD21*^+^* B cells, and NK cells (26). Contrary to human and mouse (10, 51) neutrophils, bovine neutrophils do not seem to express CLEC7A (52). Thus, although bovine neutrophils respond to Zymosan by increasing ROS production, this effect was dependent on Ca^+^ influx and mediated, at least in part, by CD11b (52), a component of CR3, an important β-glucan receptor in human neutrophils (53). Bovine-derived macrophages also increased the production of ROS in response to Zymosan, although the receptor involved was unraveled (54). In that line, a bovine macrophage cell line (BOMAC) challenged with *S. cerevisiae* cell wall components consistently expressed higher levels of IL-6, regardless of the yeast strain used, but no confirmation of the receptor responsible for cell activation nor evaluation of putative β-glucan-receptors’ expression were done (55). Nevertheless, a human fibroblast cell line (HEK293) transfected with boDectin-1 responded to Zymosan by increasing the production of IL-8, indicating that this bovine receptor could directly recognize β-glucan-containing particles (37).

Here we demonstrated that bovine monocytes respond to particulate β-glucans, through Dectin-1 triggering, resulting in increased expression of pro-inflammatory cytokines. Incubation of bovine monocytes with soluble β-glucans did not induce the production and mRNA expression of any of the cytokines assessed, nor the expression of MHC class II and costimulatory molecules CD80 and CD86 on the surface of monocytes, suggesting that soluble β-glucans do not activate bovine CLEC7A *in vitro*. Soluble β-glucans, despite being ligands of human and murine Dectin-1, are not able to cluster and activate *in vitro* the receptor (16), thus not inducing downstream cell signaling and activation (56). Pro-inflammatory cytokine production and cytokine gene expression were significantly increased in cells stimulated with dispersible β-glucans and Zymosan in a dose-dependent manner. These results are consistent with *in vitro* data obtained with murine bone marrow-derived macrophages and dendritic cells (57), murine resident macrophages (56), human whole blood (58), and porcine innate immune cells, namely peripheral-blood mononuclear cells and neutrophils (59). Cytokine response was in accordance with the increased costimulatory molecule expression observed on the surface of WGP-Dispersible and Zymosan-treated monocytes. Dispersible β-glucans and Zymosan, induced the upregulation of costimulatory molecules, which might contribute to improve T cell stimulation. Other authors (12, 14) have previously highlighted the importance of a crosstalk between different receptors, such as Dectin-1 and TLR-2. When several PRRs are activated simultaneously by particulate β-glucans, a complex cascade of cell signaling is usually amplified by this collaboration (60). Both Dectin-1 and TLR-2 recognize Zymosan, thus Zymosan is likely able to induce a more sustained and marked cell stimulation with a concomitant higher cytokine production.

The positive correlation found here between *CLEC7A* expression and *TNF* and *IL6* expression and IL-8 production in cells stimulated with WGP-Dispersible indicates that BoDectin-1 mediates β-glucan recognition in bovine monocytes. We found, however, no correlation between *CLEC7A* expression and cytokine production or gene expression in Zymosan stimulated cells. Since Zymosan contains other pathogen-associated molecular patterns besides β-glucans, other receptors being triggered by those compounds could be contributing to cytokine production and hamper a direct association. Although a combined recognition of Zymosan by multiple PRRs, was reported in human and mouse cells, namely by Dectin-1 plus TLR-2 (61), Willcocks et al. (37) have reported that HEK293 cell line transfected with both boDectin-1 and boTLR-2 did not increase the production of IL-8 in response to Zymosan comparatively to HEK293 cells expressing boDectin-1 alone. Indeed, in that particular study, HEK293-boTLR-2 did not respond to Zymosan (37).

We found a negative correlation between *CLEC7A* and *IL10* gene expression, reinforcing the ability of this highly pure β-glucan in inducing the production of pro-inflammatory cytokines, rather than anti-inflammatory cytokines. In contrast, Zymosan at 100 µg/mL induced the expression of the anti-inflammatory cytokine IL-10, comparatively to control cells. This is in accordance with previous reports describing an increased production of IL-10 in human and murine dendritic cells in response to these β-glucan-containing particles, which may confer immunological cell tolerance (62). Since WGP-Dispersible, which does not trigger TLR-signaling, did not induce *IL10* gene expression, we hypothesize that TLR-2, a receptor already associated with high IL-10 production (63), is being activated upon recognizing other Zymosan components.

These results indicate that boDectin-1 on bovine monocytes is effectively being triggered by particulate β-glucans similarly to mouse and human monocytes. In contrast, in porcine macrophages, Dectin-1 silencing did not affect cytokine production (38), reinforcing the species-specific nature of the PRR response to several agonists (37, 54). We have attempted Dectin-1 blockade with an anti-human Dectin-1 neutralizing mAb without success. The availability of a bovine anti-Dectin-1 mAb, with neutralizing functions, would be of most importance to both assess cell surface Dectin-1 expression and perform further functional assays. This would more directly allow to uncover the signaling pathways elicited by particulate β-glucans. Since Dectin-1 knockdown using siRNAs did not completely resume IL-8 production and *TNF*, *IL6*, and *IL1B* expression to negative control levels, it is possible that other receptors could additionally be involved in the recognition of WGP-Dispersible. It thus remains to be elucidated whether bovine CR3 is also playing a role on the recognition of particulate β-glucans by bovine monocytes, as in human monocytes (64) and in mouse (65) and swine macrophages (38).

Recognition of β-glucans by Dectin-1 has been shown to induce epigenetic modifications in immune cells, that render them more efficient in responding to infection (66), a phenomenon referred to as trained immunity (66, 67). It is therefore conceivable that bovine monocytes, such as those of mice (68), humans (28), dogs (69), and chicken (70) may be prone to induction of trained immunity. That would provide a plausible explanation for the beneficial effects of β-glucan-containing dietary supplements observed *in vivo* in cattle (23, 45–47). The *in vitro* results obtained in this study may help clarify β-glucan recognition by bovine monocytes and lend support to further studies addressing trained immunity events in this species.

## Data Availability Statement

The raw data supporting the conclusions of this article will be made available by the authors, without undue reservation.

## Author Contributions

AP conducted the experiments, performed data acquisition and analysis, and wrote the manuscript. AC, TL, RF-M, and BL participated in the experiments. EM and IR assisted in the design of the experiments. ARJC and AF assisted in the interpretation of data. AC, MM, and MV conceived and designed the experiments, supervised the experimental work, assisted in data acquisition and analysis, and assisted in manuscript writing. All authors contributed to the article and approved the submitted version.

## Funding

This work received financial support from PT national funds (FCT/MCTES, Fundação para a Ciência e Tecnologia and Ministério da Ciência, Tecnologia e Ensino Superior) through the project UIDB/50006/2020. AP was supported by FCT phD grant PD/BDE/135540/2018. AC was supported by FCT Individual CEEC 2017 Assistant Researcher Grant CEECIND/01514/2017. MM was supported by FCT through program DL 57/2016 – Norma transitória (SFRH/BPD/70716/2010).

## Conflict of Interest

The authors declare that the research was conducted in the absence of any commercial or financial relationships that could be construed as a potential conflict of interest.

## Abbreviations

B2M, β2 Microglobulin; CD, Cluster of Differentiation; cDNA, Complementary DNA; CLEC7A, C-Type Lectin Domain Containing 7A; CR3, Complement Receptor 3; DAPI, 4′,6-diamidino-2-phenylindole; ELISA, Enzyme-Linked Immunosorbent Assay; FACS, Fluorescence-activated Cell Sorting; FITC, Fluorescein isothiocyanate; FMO, Fluorescence Minus One; FVD, Fixable Viability Dye; IgG, Immunoglobulin G; IL, Interleukine; LDH, Lactate dehydrogenase; LPS, Lipopolysaccharide; mAb, monoclonal Antibody; MARVELD1, Marvel Domain Containing 1; MHC, Major Histocompatibility Complex; NCBI, National Center for Biotechnology Information; P3C, Pam3CSK4 [Pam3Cys-Ser-(Lys)4]; PerCP-Cy5.5, Peridinin-chlorophyll protein-cychrome 5.5; PRR, Pattern Recognition Receptors; RNA, Ribonucleic Acid; ROS, Reactive Oxygen Species; RPE, R-Phycoerythrin; SEAP, Secreted Embryonic Alkaline Phosphatase; siRNA, Small Interference Ribonucleic Acid; PCR - Polymerase Chain Reaction; TAE, Tris acetate EDTA; TLR, Toll-like Receptor; TNF-α, Tumour Necrosis Factor Alpha; UNC - MISSION^®^ siRNA Universal Negative Control #1; WGP-D, Whole Glucan Particles – Dispersible; WGP-S - Whole Glucan Particles – Soluble; Zym, Zymosan.
