# Supplementary material for: Dectin-1-Mediated Production of Pro-Inflammatory Cytokines Induced by Yeast β-Glucans in Bovine Monocytes
Source: Front Immunol. 2021 May 28;12:689879. doi: 10.3389/fimmu.2021.689879 (PMC8195389; doi:10.3389/fimmu.2021.689879)
Supplement: Supplementary file 1 [file DataSheet_1.pdf]

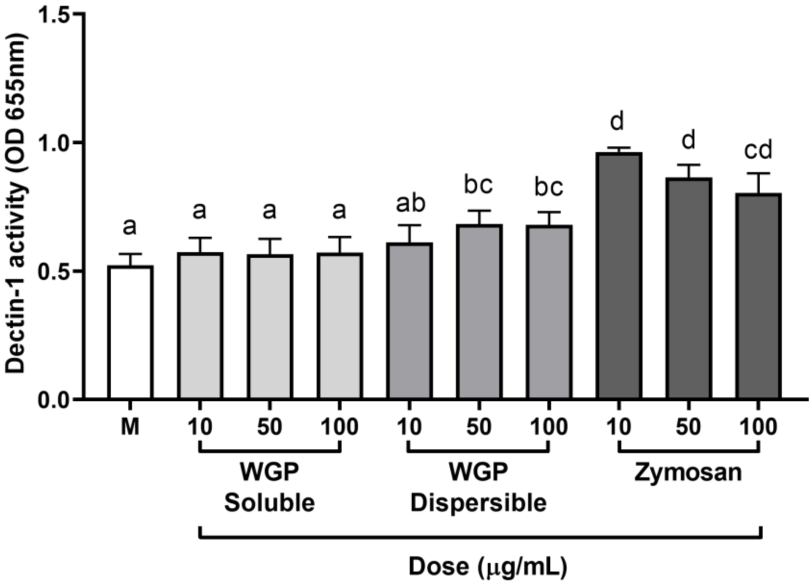

Supplementary Figure 1 - Human Dectin-1b activity measured through substract hydrolysis by secreted embryonic alkaline phosphatase (SEAP), produced upon activation of NF-κB in HEK-Blue™ hDectin-1b cells cultured for 16 h without stimulus (M) or stimulated with WGP Soluble (WGP-S), WGP Dispersible (WGP-D) or Zymosan (Zym). Data from three independent experiments performed in triplicate, displayed as means plus standard error of the mean. [a,b,c,d]Means with different superscript letters are significantly different (P<0.05).

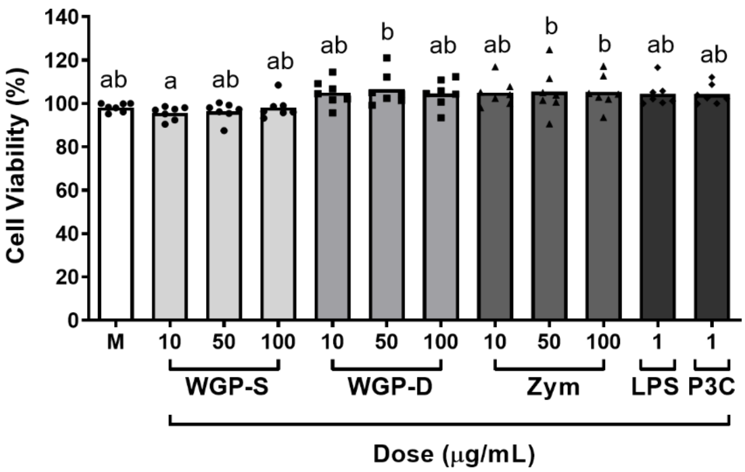

Supplementary Figure 2 - LDH released by bovine monocytes cultured for 24 h without stimulus (M) or stimulated with WGP Soluble (WGP-S), WGP Dispersible (WGP-D), Zymosan (Zym), LPS, and pam3csk4 (P3C). Bars represent means of data from seven animals. [a,b]Means with different superscript letters are significantly different (P<0.05).

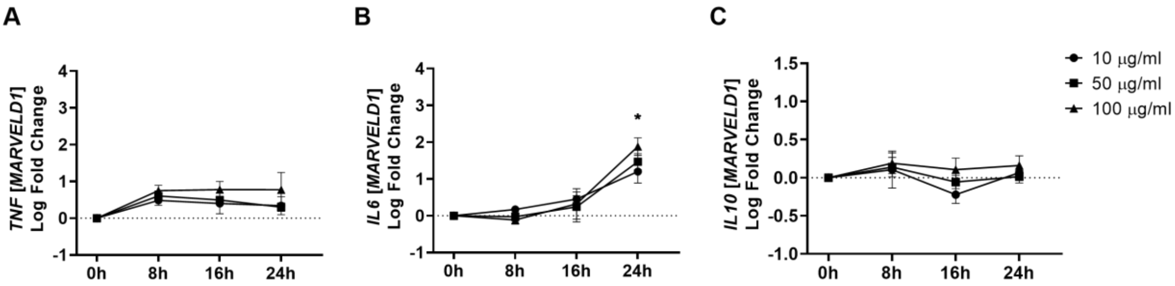

Supplementary Figure 3 - Cytokine relative mRNA expression evaluated by RT-PCR in bovine monocytes and normalized to the mRNA expression of the reference gene *MARVELD1*. Cells cultured for 8, 16, and 24 h with WGP Dispersible. Data are presented as Log fold change relative to medium (M) and represent means plus SEM of data from four animals. * *P*<0.05, relative to 0 h condition.

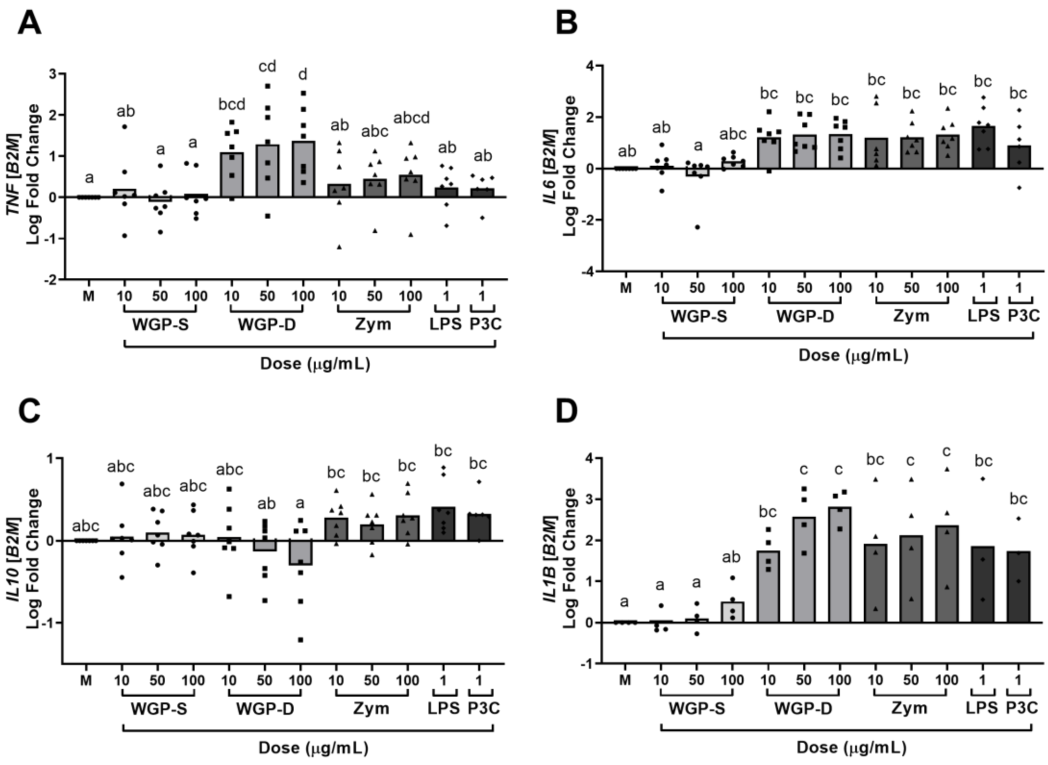

Supplementary Figure 4 - Cytokine relative mRNA expression (A, B, C) evaluated by RT-PCR in bovine monocytes and normalized to the mRNA expression of the reference gene beta-2-microglobulin (*B2M*). Cells were cultured for 24 h with WGP Soluble (WGP-S), WGP Dispersible (WGP-D), Zymosan (Zym), LPS, and pam3csk4 (P3C). Data are presented as Log fold change relative to medium (M) and represent means of data from seven animals for *TNF*, *IL6*, and *IL10*, and four animals for *IL1B*. Each symbol corresponds to an independent biological sample. [a,b,c,d]Means with different superscript letters are significantly different (*P*<0.05).

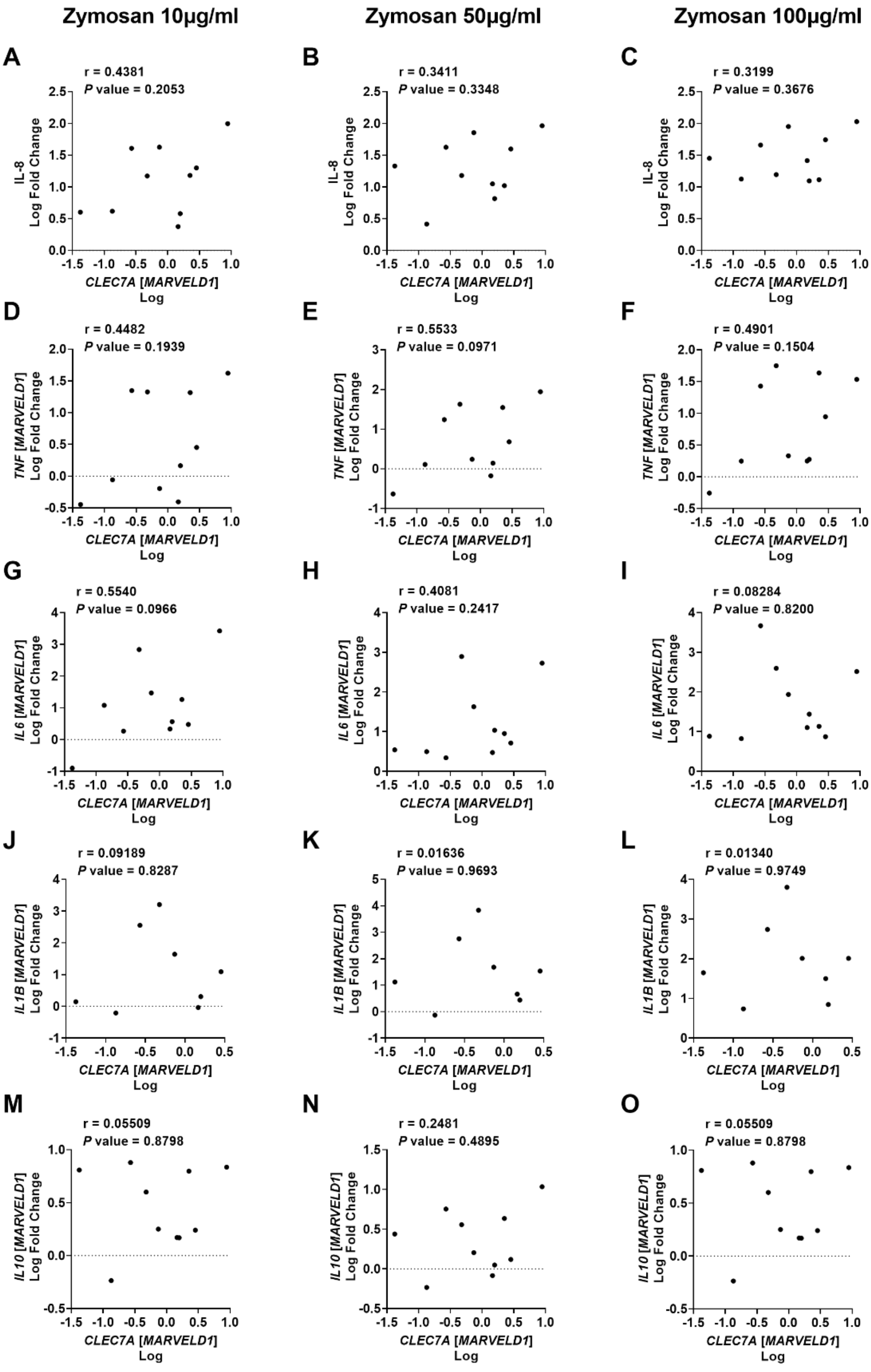

Supplementary Figure 5 - Correlations between *CLEC7A* mRNA expression and (A, B, C) IL-8 cytokine production, (D, E, F) *TNF*, (G, H, I) *IL6*, (J, K, L) *IL1B*, and (M, N, O) *IL10* mRNA expression upon stimulation with 10, 50, and 100 µg/mL of Zymosan, as indicated. Results are presented as Log fold changes of each cytokine relative to medium *vs* Log *CLEC7A* mRNA. Data represent simple linear regressions, with Pearson correlation coefficients (r) and *P* values.

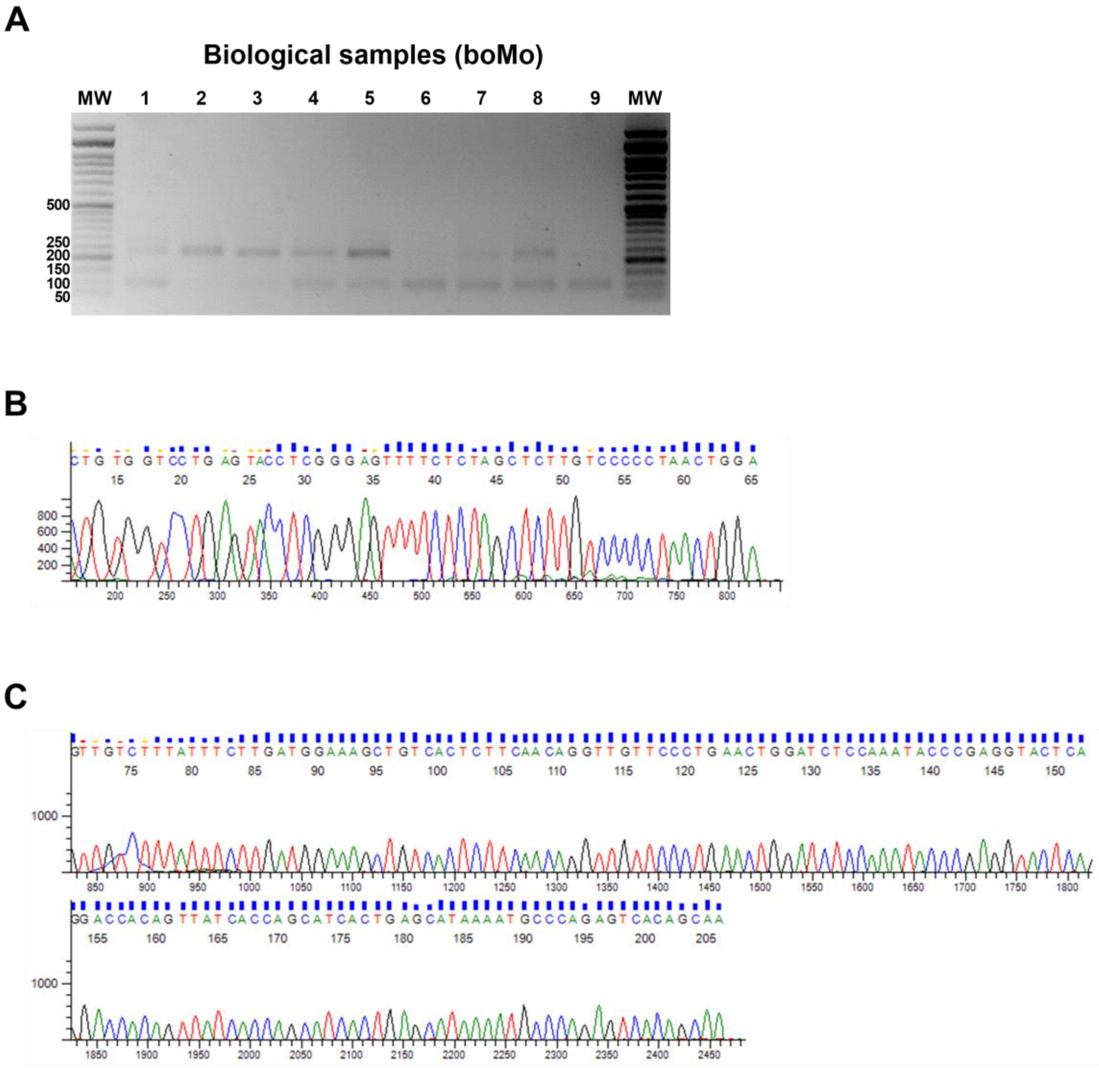

Supplementary Figure 6 - Electrophoretic profile of *CLEC7A* PCR amplification products from bovine monocytes cDNA, visualized in a 1.5% TAE agarose gel stained with ethidium bromide (A). MW - Molecular weight marker NZYDNA Ladder VI. Band sizes are shown in base pairs (bp); Representative electropherograms of (B) bovine *CLEC7A* PCR product sequencing of the smaller PCR product and (C) of the larger PCR product. The PCR products had 100% identity with Bovine *CLEC7A* mRNA sequences XM_005207062.4, XM_024991882.1, XM_005207061.4, XM_005207064.4, AY937382.1, BC102340.1, and NM_001031852.1, compared using the NCBI database and BLAST algorithm. DNA sequencing of PCR fragments was performed at the Genomics i3S Scientific Platform. PCR products were purified using illustraTMSephadexTMG-50 Fine DNA Grade according to the manufacturer’s protocol. Sequencing products were analyzed by capillary electrophoresis on a 3500 Genetic Analyzer (Applied Biosystems). Electropherograms were visualized using Sequence Scanner Software 2 v2.0 (Applied Biosystems).

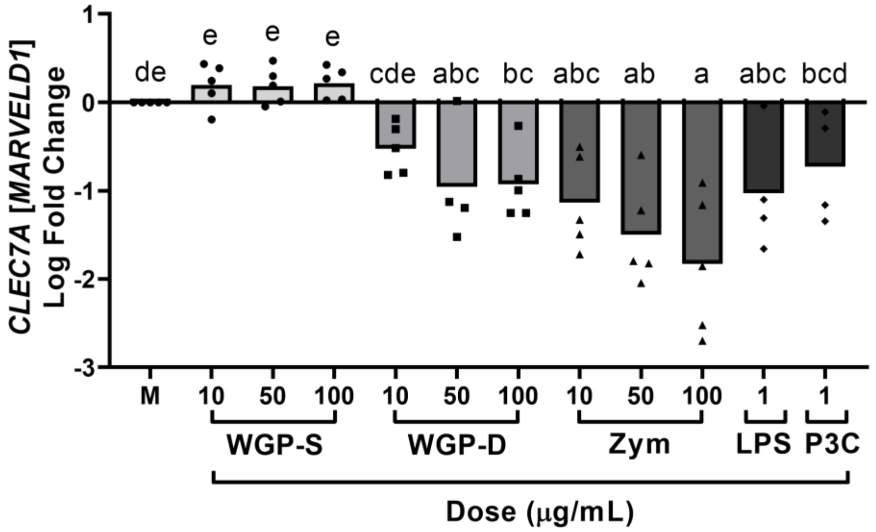

Supplementary Figure 7 - CLEC7A relative mRNA expression evaluated by RT-PCR in bovine monocytes and normalized to the mRNA expression of the reference gene *MARVELD1*. Cells were cultured for 24 h with WGP Soluble (WGP-S), WGP Dispersible (WGP-D), Zymosan (Zym), LPS, and pam3csk4 (P3C). Data are presented as Log fold change relative to medium (M) and represent means of data from five animals. Each symbol corresponds to an independent biological sample. [a,b,c,d,e]Means with different superscript letters are significantly different ($P<0.05$).

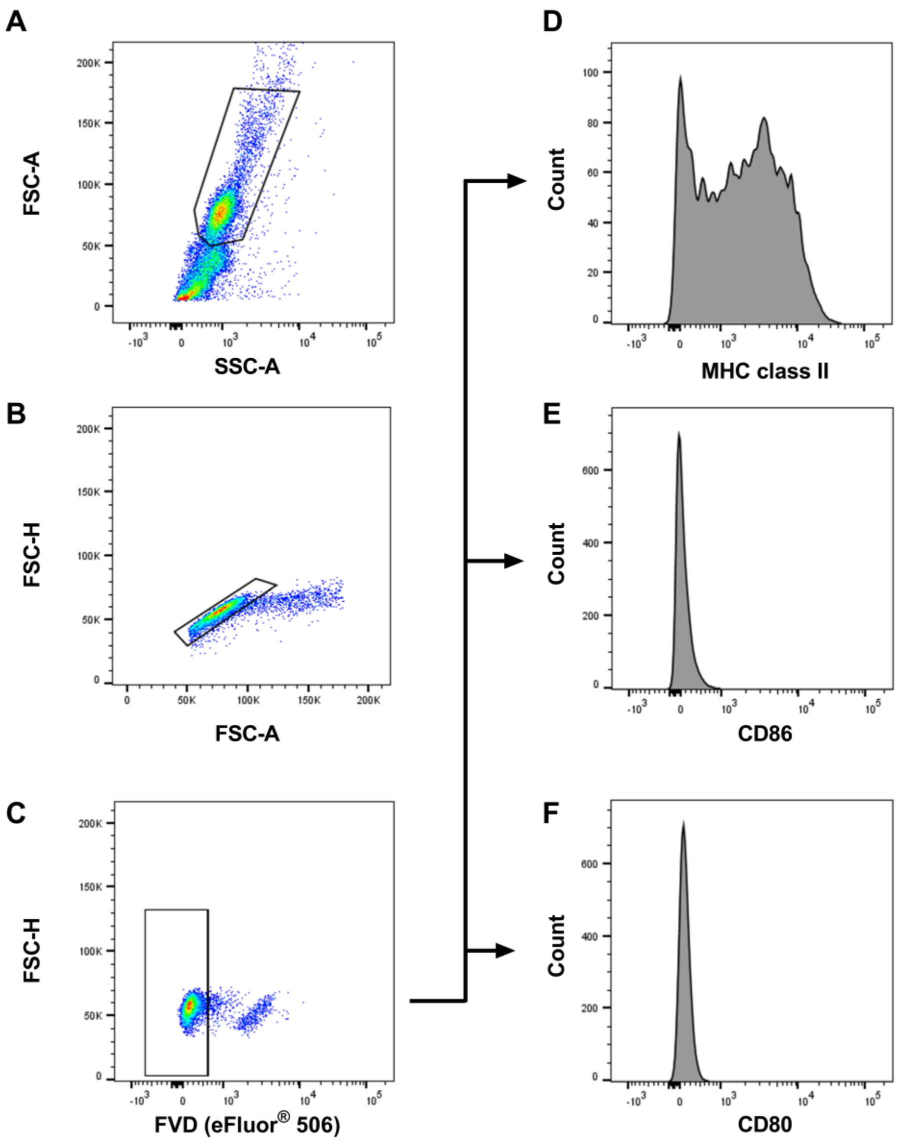

Supplementary Figure 8 - Flow cytometry gating strategy used for evaluation of cell surface co-stimulatory (CD80/CD86) and MHC class II molecule expression on bovine monocytes. Gating strategy was based on (A) exclusion of cell debris, (B) selection of single cells, and (C) exclusion of dead cells using a Fixable Viability Dye (eFluor® 506), followed by analysis of the mean fluorescence intensities due to (D) MHC class II, (E) CD86 and (F) CD80 staining. Dot plots and histograms are representative examples and correspond to bovine monocytes stimulated with for 16 h WGP Dispersible.

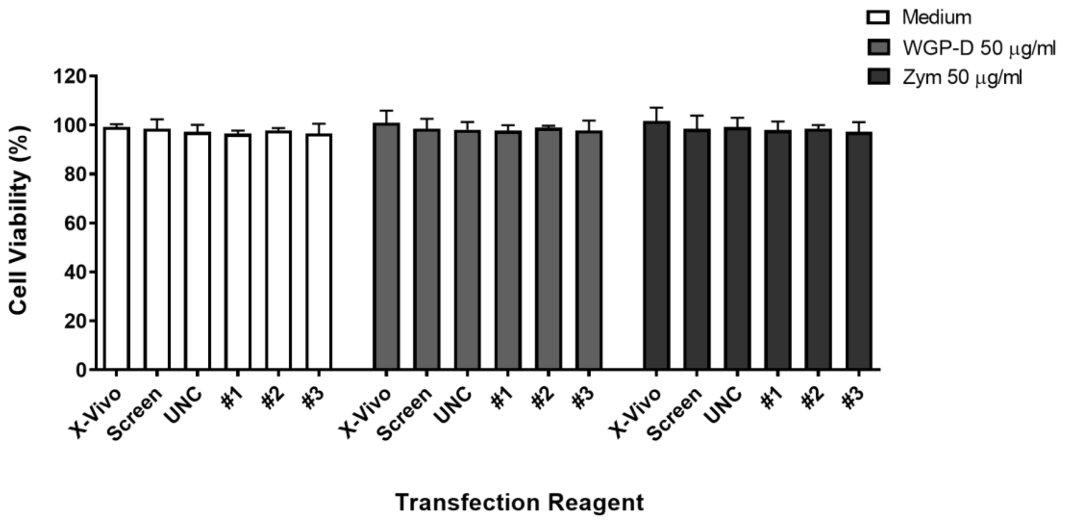

Supplementary Figure 9 - LDH released by bovine monocytes treated with serum-free X-Vivo™medium (X-Vivo), transfection reagent medium (Screenfect), MISSION® siRNA Universal Negative Control #1 (UNC) and three different siRNA duplexes targeting bovine Dectin-1 mRNA (siRNA #1, siRNA #2 and siRNA #3). After 4 hours of transfection, cells were stimulated WGP-Dispersible (WGP-D) and Zymosan (Zym) at 50 µg/mL or medium (M), for 24 hours. Results are presented as percentage of live cells and correspond to means plus standard error of the mean from three different animals.

Supplementary Table 1 - Bovine Dectin-1 mRNA expression (E), normalized to MARVELD1, of cells treated with serum-free X-Vivo™medium, transfection reagent medium (ScreenFect® siRNA), MISSION® siRNA Universal Negative Control #1 and three different siRNA duplexes targeting bovine Dectin-1 mRNA (siRNA #1, siRNA #2 and siRNA #3).

Transfection Reagent	Biological Sample #1	Biological Sample #2	Biological Sample #3
X-Vivo™medium	1,067	1,448	1,138
ScreenFect® siRNA	0,179	0,412	0,911
Universal Negative Control	0,127	1,044	1,391
siRNA duplex #1	0,081	1,115	1,501
siRNA duplex #2	0,088	0,204	0,154
siRNA duplex #3	0,010	0,204	0,229

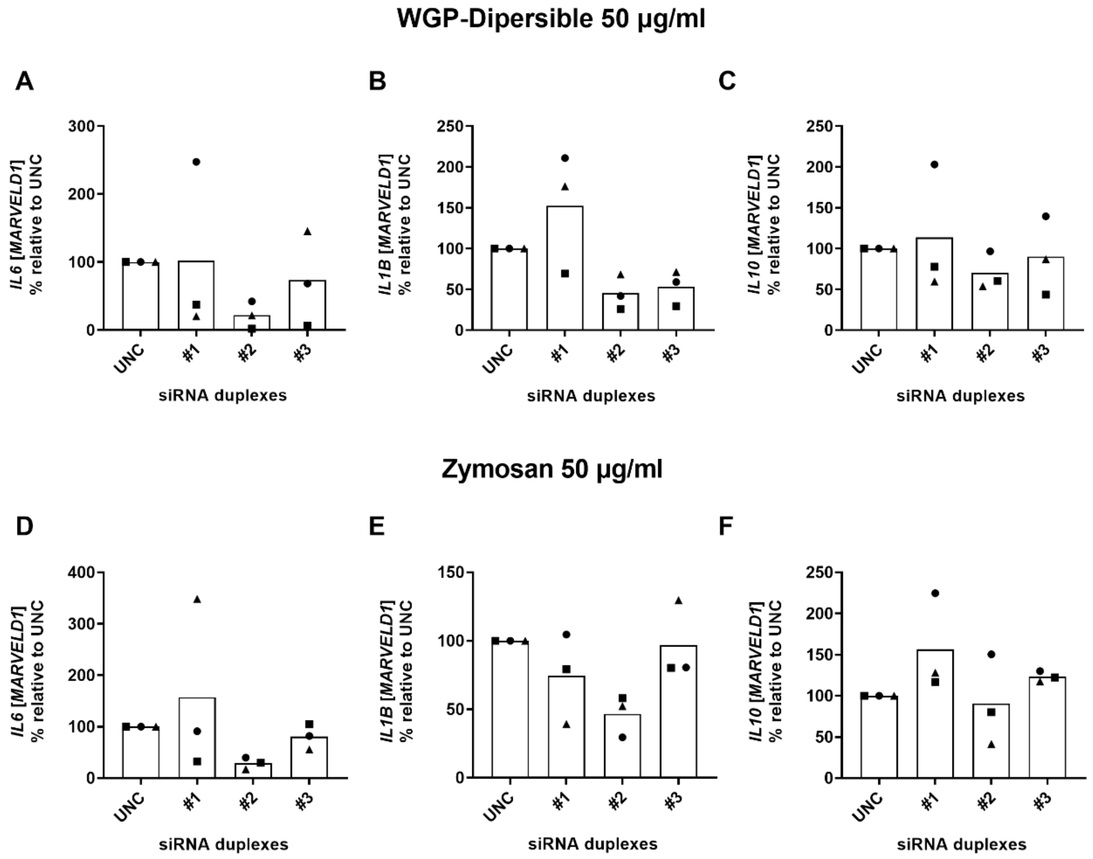

Supplementary Figure 10 - *IL6* (A and D), *IL1B* (B and E), and *IL10* (C and F) mRNA expression of cells transfected with duplexes #1, #2 and #3 and MISSION® siRNA Universal Negative Control #1 (UNC) and stimulated with WGP-Dispersible (A, B and C) or Zymosan (D, E, and F) at 50 µg/mL, calculated in percentual change relative to UNC transfected cells. Results correspond to means from three different animals (each one represented by squares, triangles or circles).

A

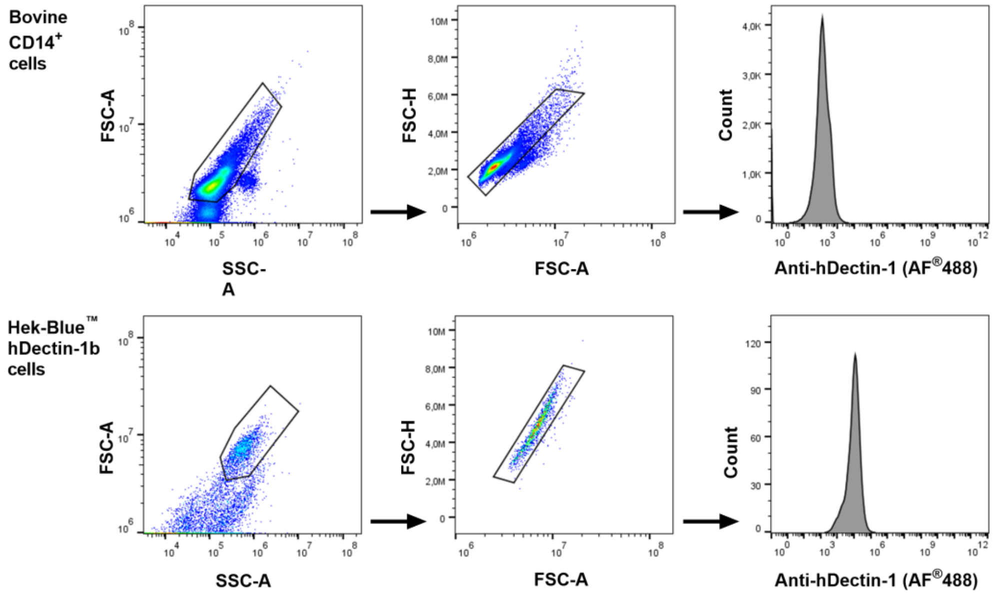

B

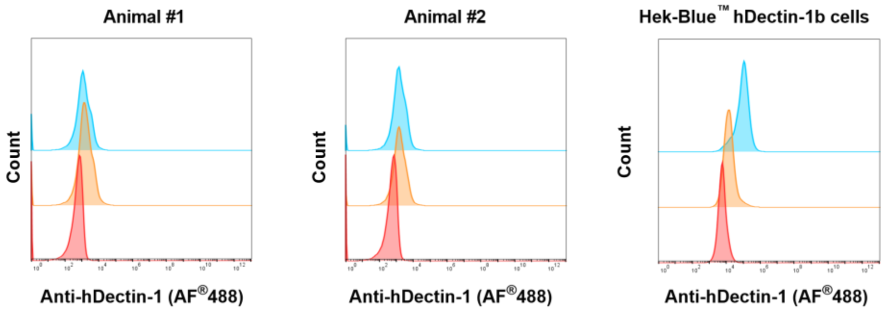

C

Samples	Mean Fluoresce Intensities		
	Unstained	Secondary Antibody	Anti-hDectin-1 plus Secondary Antibody
Animal #1	574	3 949	2 186
Animal #2	573	3 346	3 030
Hek-Blue™ hDectin-1b Cells	7 172	27 007	122 260

Supplementary Figure 11 - Flow cytometry gating strategy (A) used for cell surface detection of Dectin-1 on bovine CD14$^+$ cells (monocytes) and on HEK-Blue™ hDectin-1b cells. Gating strategy was based on exclusion of cell debris, followed by selection of single cells and analysis of Dectin-1 expression. (B) Flow cytometry histogram overlays of bovine CD14$^+$ cells from two different animals and HEK-Blue™ hDectin-1b cells stained with anti-human Dectin-1/CLEC7A antibody followed by incubation with a secondary antibody conjugated with Alexa-Fluor® 488. Histograms in red correspond to unstained samples, in orange to samples incubated only with the secondary antibody, and in blue samples incubated with primary and secondary antibodies. (C) Mean Fluorescence Intensities obtained by flow cytometry analysis of bovine CD14$^+$ cells from the two different animals and HEK-Blue™ hDectin-1b cells.

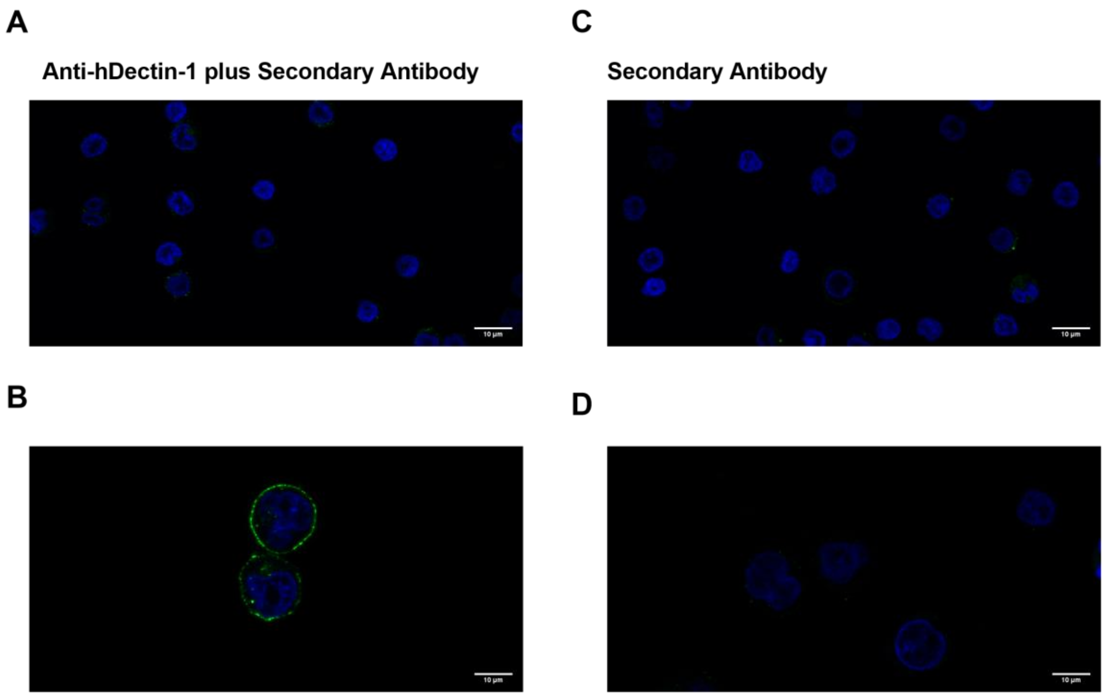

Supplementary Figure 12 - Confocal Microscopy imaging of (A) bovine monocytes (Animal #1) and (B) HEK-Blue™ hDectin-1b cell line labelled with anti-human Dectin-1/CLEC7A antibody and secondary goat anti-mouse IgG conjugated with Alexa-Fluor 488. (C) Bovine monocytes and (D) HEK-Blue™ hDectin-1b cell line labelled with secondary goat anti-mouse IgG conjugated with Alexa-Fluor® 488 only, to detect unspecific binding. Blue: nuclei stained with DAPI; Green: Dectin-1 labelled with anti-human Dectin-1/CLEC7A antibody and secondary goat anti-mouse IgG conjugated with Alexa-Fluor 488.
